# Re: Toxic metals in Loggerhead sea turtles (*Caretta caretta*) stranded freshly dead along Sicilian coasts

**DOI:** 10.1080/01652176.2023.2205491

**Published:** 2023-05-03

**Authors:** Catarina Jota Baptista, José M. Gonzalo-Orden, Fernanda Seixas, Paula A. Oliveira

**Affiliations:** a Departamento de Ciências Veterinárias, Escola de Ciências Agrárias e Veterinárias (ECAV), Universidade de Trás-os-Montes e Alto Douro, UTAD, Vila Real, Portugal; b Centro de Investigação das Tecnologias Agroambientais e Biológicas (CITAB), UTAD, Vila Real, Portugal; c Instituto de Biomedicina (IBIOMED), Universidad de León, León, España; d Faculdade de Medicina Veterinária, Universidade Lusófona de Humanidades e Tecnologias, Lisboa, Portugal; e Centro de Ciência Animal e Veterinária (CECAV), Al4Animals, UTAD, Vila Real, Portugal

**Keywords:** Metal, metalloid, wildlife

We would like to express our scientific opinion on the recently published article entitled ‘Toxic metals in Loggerhead sea turtles (*Caretta caretta*) stranded freshly dead along Sicilian coasts’ by Cammilleri et al. 2022 (Veterinary Quarterly 43(1):1-17). Considering the lack of guidelines and references that have been established for biomonitoring heavy metals and metalloids in wildlife, it is undoubtedly important to critically analyse the published studies to define aims and standard procedures for these assessments. Moreover, heavy metal(loid)s pollution is a current One Health serious concern (Zaynab et al. 2022).

This article represents an illustration of how toxic substances (as heavy metal(loid)s) can be analysed and interpreted with epidemiology. Using a long-living species, the Loggerhead sea turtle (*C. caretta*), these authors accessed for As, Cd, and Pb accumulation in adipose and muscle tissues. A modified K index suggested a good body condition. Overall, their results on muscle and adipose tissues suggest chronic relevant exposure to Pb which might negatively affect these animals’ health.

From our point of view, this heavy metal(loid)s study deserves a positive spotlight. Considering the aim of assessing chronic exposure, it is vital to relate the metal(loid) determinations with biological parameters that allow us to figure out what happens during the specimens’ life, if exposed to metal(loid). Most authors (especially considering studies with avian and mammalian species) use other parameters, such as body weight (Jota Baptista et al. 2023). These authors used Fulton’s K index, which provides a more accurate estimate of growth and body condition during the animal’s lifetime, especially considering fish and reptiles’ specific metabolism. Moreover, both males (*n* = 25) and females (*n* = 15) were sampled and tested for significant differences, further refining this approach. Thus, this article demonstrates a logical and consistent methodology, adapted to the studied species.

Another positive aspect is the way it tries to figure out the local causes for the pollutants’ values. As an illustration, the authors discuss the values of Pb, based on the sources present on the Sicilian coast. Even if it may be considered a vague hypothesis, it is useful to express this rational thinking in the Discussion sections of these articles. With this, it would be possible to design new studies and build new conservation and research projects to clarify the real causes of these problems.

In contrast, we would like to express our perspective on the type of samples used in this assessment. Considering the expansion of the use of non-invasive samples and the nesting spot that Sicily is, eggshells of hatched or unhatched turtle eggs could also be used to access metal(loids), as non-invasive samples. On the other hand, considering invasive sampling, the opportunity to perform necropsies gives access to a variety of tissues (including the liver or the kidneys), which are described as more suitable to analyse some metal(loid)s, such as Cd. Moreover, histopathology could have provided crucial information on the tissue effects of these compounds (as Pb), clarifying their deleterious impact (Jota Baptista et al., 2022).

Finally, we believe every wildlife study on environmental pollution should always provide a One Health approach discussion and provide the reasons and criteria for choosing a bioindicator or sentinel species. Besides long-living, the species distribution, abundance and evident changes when exposed to these substances should be part of these criteria. Heavy metal(loid)s pollution has repercussions on the health of humans, animals and ecosystems in a way that is impossible to prioritize one of these three closely related branches (Jota Baptista et al., 2022). Therefore, considering the importance that One Health should have for veterinarians and veterinary actions, we would have suggested a brief consideration. Notwithstanding, specific assessments to verify if chronic exposure to these chemical substances compromises the health and populational stability of a certain species are also relevant, especially for conservation purposes.

In conclusion, ‘Toxic metals in Loggerhead sea turtles (*Caretta caretta*) stranded freshly dead along Sicilian coasts’ provided our community with a brief, clear and objective study of metal(loid)s exposure of *C. caretta,* a consistent example of how veterinary epidemiology and environmental sciences can be allied to find problems’ origins and possible solutions.
